# Dataset of pulse – echo ultrasonic a – scan signals for derived recovery features of NMC – 622 cathode slurries during post – mixing structural evolution

**DOI:** 10.1016/j.dib.2026.112795

**Published:** 2026-04-24

**Authors:** Erdogan Guk, Hamidreza Farhadi Tolie, Mona Faraji Niri, James Marco

**Affiliations:** a Warwick Manufacturing Group (WMG), University of Warwick, Coventry CV4 7AL, United Kingdom; b The Faraday Institution, Harwell Science and Innovation Campus, Didcot OX11 0RA, United Kingdom

**Keywords:** Ultrasonic testing, Lithium-ion battery manufacturing, Cathode slurry, Structural recovery, Process monitoring, Production line parameterisation

## Abstract

This data article presents an open ultrasonic pulse – echo dataset acquired from NMC – 622 cathode slurries monitored under static conditions to characterise their post – mixing structural recovery behaviour. Slurries were prepared at varying solid contents of 63, 65, 67 and 69 wt.% and transferred into a fixed test container for ultrasonic monitoring. A 5 MHz contact transducer was used to record continuous A – Scan waveform under consistent acquisition settings (raw time – domain signals paired with frequency – domain representation). From each waveform, reflection amplitude and time – of – flight (ToF) features extracted from the slurry – air interface echo provides a compact representation of evolving acoustic response over time. The dataset is intended for benchmarking of signal processing pipelines, development of physics – informed feature extraction, and machine – learning models for slurry metrology and coating readiness assessment. By Packing signals, minimal metadata, and lightweight visualisation and analysis script, the resources enable reproducible reuse across materials – processing and signal – processing communities.

Specifications Table

**Table:** 

Subject	Engineering & Materials science
Specific subject area	Ultrasonic metrology for lithium-ion battery cathode slurry recovery and ageing (post – mixing evolution).
Type of data	Raw ultrasonic A – scan waveforms (time domain) and corresponding frequency – domain representations (e.g., Fast Fourier Transform (FFT) magnitude spectra). Supporting code is provided for data loading and visualisation (e.g., plotting signal amplitude versus time – of – flight and basic frequency – domain plots) to facilitate reuse and independent processing.
Data collection	Pulse – echo ultrasonic testing using an EPOCH 650 flaw detector (pulser – receiver) with a 5 MHz contact microdot probe in a fixed – container configuration; waveform acquisition via custom Python interface.
Data source location	Warwick Manufacturing Group (WMG), University of Warwick, Coventry CV4 7AL, United Kingdom.
Data accessibility	Repository name: Open NMC-622 Slurry Dataset: Ultrasound Measurements Data identification number: 10.17632/vhdf9shds2.2 Direct URL to data: https://data.mendeley.com/datasets/vhdf9shds2/2 Use the DOI or URL link to access the repository, which contains the *slurry_dataset.h5* file with ultrasound measurements for all solid content samples. Start with the provided Readme file to understand the dataset description, file structure, variables, and units. Cite the dataset DOI whenever reusing the data. In addition to the dataset file, the repository includes: • A dedicated folder contains Python scripts for loading the dataset, visualising the time – and frequency – domain signals, and performing detailed analyses. These analyses include trend change detection algorithms to segment signal evolution and unsupervised machine learning models, such as K – means, to cluster patterns in the data. In addition, a graphical interface integrates all these functionalities and provides the ability to export selected data to Excel sheets, enabling users to interactively explore and analyse the dataset. Comprehensive instructions for using both the scripts and the interface are included in an accompanying README file, ensuring reproducibility and facilitating ease of use for both novice and advanced users. • A requirements.txt file specifying the required Python library versions. The slurry_dataset.h5 file contains all samples in a single structured hierarchy, including raw ultrasound signals, backwall echoes, and FFT-derived features for each sample.
Related research article	E. Guk, H. Farhadi Tolie, P. Bellchambers, M. Capener, M. Faraji Niri, and J. Marco, “Ultrasonic characterisation of lithium-ion battery cathode slurries: Monitoring post-mixing evolution and structural recovery,” *Journal of Energy Storage*, submitted for publication (Under Review): Manuscript Number: EST-D–25–16643R1

## 1 Value of the Data

• **Importance**
  The introduced open-access ultrasonic pulse – echo dataset is obtained by following the standard protocols for lithium – ion battery slurry manufacturing, capturing the post – mixing structural recovery and early ageing of NMC – 622 cathode slurries under static conditions. The dataset comprises raw A – scan waveforms (time domain) and their frequency – domain representations derived via FFT, measured across solid contents of 63, 65, 67 and – 69 wt.%, i.e., a practically relevant range around standard baseline of 65 wt.%.
  Ultrasonic monitoring at the slurry stage has not been presented yet to author best knowledge despite its potential value for manufacturing metrology as a non – destructive technique. Compared with the standard testing such as electrochemical impedance spectroscopy (EIS) and electrical impedance tomography (EIT), ultrasonic monitoring offers practical advantages especially for slurry and electrode manufacturing [1]. It can track the mechanical and microstructural properties without electrical contact, making it well suited to highly concentrated and dense slurries [[2], [3], [4]]. In addition, ultrasonic sensing enables rapid, non-destructive tracking of structural changes such as particle packing, density variations, and recovery behaviour through changes in wave propagation [5]. In this dataset, a pulse – echo configuration records distinct reflection groups across varying slurry solid contents, enabling re – analysis of amplitude – and time – of – flight – driven behaviours associated with recovery processes such as particle redistribution, binder network rebuilding, densification, and sedimentation trends.
  To maximise reusability, the data package is curated in a reproducible form and is accompanied by a lightweight Python script for loading and visualising the signals (e.g., amplitude vs time – of – flight plots and frequency – domain spectra), allowing rapid inspection and independent downstream processing without requiring proprietary software. The emphasis is on providing recognised, analysis-ready raw signals (time and frequency domains), rather than reproducing or constraining interpretation to a single feature extraction pipeline.
  While the ultrasonic acquisition originates from the accompanying slurry study describing recovery and ageing behaviour, the present dataset release is distinguished by (I) Providing the complete raw A – scan signal sets (not only derived trends), (II) Including harmonised frequency – domain versions to support spectral benchmarking, (III) Offering reusable visualisation code that enables consistent inspection across solid contents and time points, (IV) Including script for trend – change detection in the time-domain signals, and (V) Including script for clustering analysis of frequency-domain signals.
• **Target Audience**
  Researchers in battery manufacturing specifically for those working on the metrology of slurries including its processability, structural recovery, and storage stability before coating in NMC – class formulations. Ultrasonic/signal – processing researchers developing robust time – domain and spectral analysis methods and seeking data for signal attenuating, due to heterogeneous suspensions of the slurries. Data science and machine – learning experts developing models (can be good data to be used for verification of other models as well as future generation) for slurry state estimation, monitoring, and future digital – twin frameworks using continuous acoustic signals as inputs. Industrial engineers seeking non – invasive sensing approaches for quality assurance of slurry preparation and coating readiness (e.g., detecting abnormal recovery kinetics, instability, or sedimentation onset).
  The dataset can be primarily used for optimisation of the inspection/manufacturing parameters to industrial level scale – up. Besides, it can also support development and validation of predictive models linking ultrasonic features with slurry rheological behaviour, including viscosity evolution during structural recovery within the investigated solid-content range (63–69 wt.%), as discussed in the associated research article.
• **Future Use**
  The dataset enables systematic study of how solid content affects ultrasonic signal evolution during post – mixing recovery, supporting both time – domain and frequency – domain modelling approaches. Users can benchmark methods for: (i) time – domain analysis (echo identification, amplitude tracking, ToF estimation, denoising), and (ii) spectral analysis (dominant band shifts, broadband attenuation trends, spectral energy redistribution) using the shared FFT representations.
  The raw signals can be reused to develop and compare algorithms for detecting recovery stages – rapid restructuring, consolidation, and plateau as well as for identifying longer-term stability loss mechanisms such as sedimentation or agglomeration, which are challenging to capture continuously with conventional techniques. Trend change detection algorithms can be applied to track signal evolution over time by analysing the first peak amplitude trend in time – domain, corresponding to different recovery stages. In addition, unsupervised machine learning (ML) models such as K – Means can be applied directly to the frequency – domain signals to cluster these stages. Preliminary results from both approaches demonstrate the potential and practical utility of the dataset.
  By releasing raw ultrasonic A – scan time series alongside their frequency-domain (FFT) counterparts and a simple plotting utility, this dataset enables non – contact assessment of how NMC – 622 solid content influences recovery behaviour. It supports reproducible ML research for slurry metrology, including slurry state clustering, recovery time prediction, and anomaly detection for variability in sample recipes. The data can also serve as an input stream for digital – twin development and predictive control of slurry preparation and downstream coating operations.

## 2 Background

Ultrasonic testing (UT) has been started to be recognised non – destructive method in battery diagnostics where offers routes to track evolving structure in battery materials, yet manufacturing – stage applications – particularly at the slurry preparation stage – remain comparatively scarce. This gap matters because cathode slurries undergo rapid post – mixing recovery (particle redistribution and carbon – binder network rebuilding) and slower ageing (sedimentation/agglomeration) under static conditions, while conventional rheology can struggle to capture long – term evolution without artefacts from drying/gelation or imposed shear.

Building on our earlier electrode – focused ultrasonic studies and Data in Brief release [[6], [7], [8], [9]], the present work extends UT to NMC – 622 slurry monitoring using a pulse – echo configuration in a fixed container, where reflections from the cup bottom and slurry – air interface yield time – resolved signals sensitive to impedance, attenuation and sound – speed changes during recovery. Together with the previous work, it offers a complementary ultrasonic tracking of battery manufacturing process. The accompanying dataset provides raw A – scan waveforms (time domain) and corresponding frequency – domain (FFT) data across 63 – 69 wt.% solid content, together with a simple Python utility for visualisation (e.g., amplitude vs time – of – flight and spectra plots), enabling reproducible benchmarking of signal – processing approaches and supporting future data – driven slurry metrology and digital – twin development.

The dataset contains ultrasonic pulse – echo measurements acquired from NMC – 622 cathode slurries with varying solid content during post – mixing recovery – and, where relevant, during subsequent resting – under static conditions. For each slurry state, the raw time – domain A – scan waveforms are provided alongside their corresponding frequency – domain representations, obtained via an FFT. Taken together, these paired signals form a coherent and reusable resource for examining how slurry formulation, particularly variation their solids content, and resulted recovery behaviour is reflected acoustically. They also offer a convenient benchmark for developing and comparing signal – processing approaches, whether these operate directly on the time – domain echoes or on the associated spectral characteristics such as attenuation or energy redistribution.

## 3 Data Description

**Fig. 1:**
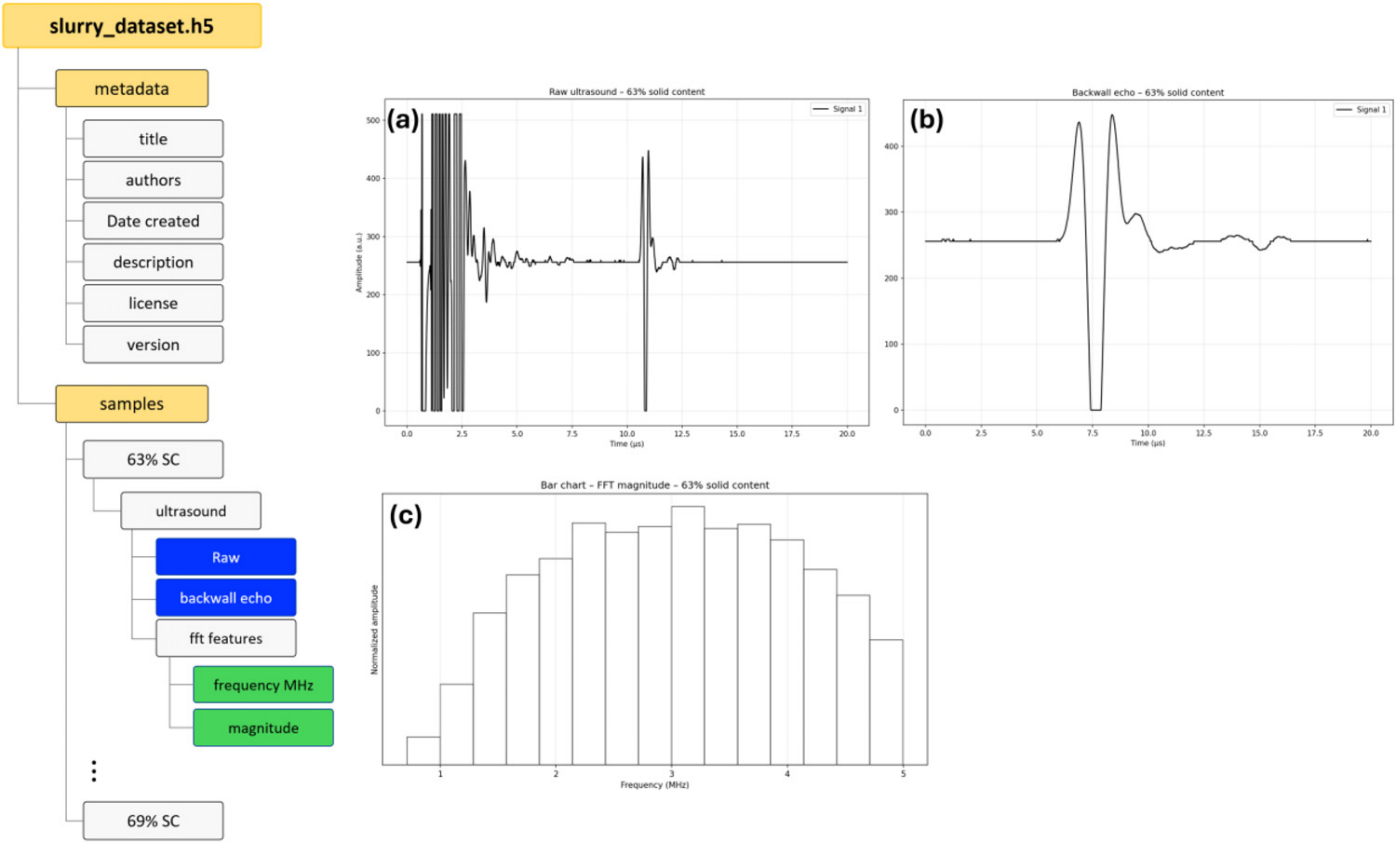
Structure of the ultrasound dataset recorded from NMC – 622 slurries. Subfigures (a) – (c) show the raw time – domain ultrasound signal, the extracted backwall echo segment, and the frequency – domain representation of the backwall echo, respectively. These representations correspond to the first frame (out of 500 ultrasound frames recorded over 1 h) for the slurry with 63% solid content.

Fig. 1 illustrates the structure of the ultrasound dataset stored in the HDF5 (.h5) format, which enables organised storage and straightforward reuse of the data. The shared dataset file (*slurry_dataset.h5*) contains two main groups: metadata and samples. The metadata group stores general dataset information, including the title, authors, creation date, description, licence, and version. The samples group contains the experimental measurements organised by slurry condition, specifically according to the solid content (i.e., 63% SC to 69% SC).

Within each solid – content group, an ultrasound subgroup stores the signal data in multiple representations. These include the Raw ultrasound A – scan signals and the extracted backwall echo signals (corresponding to the time window of approximately 9.5 – 13 µs) (Fig. 1 -a, -b). In addition, the *fft_features* subgroup provides the frequency – domain representation of the backwall echo (Fig. 1 -c). The FFT magnitude is computed and restricted to the frequency range of 0.75 – 5 MHz, where 5 MHz corresponds to the acquisition frequency limit of the recorded signals. The FFT spectrum is then trimmed to this frequency band, and the magnitude values are normalised by their maximum value. The resulting dataset stores the corresponding frequency vector (in MHz) together with the normalised FFT magnitude values. This hierarchical structure ensures that each slurry condition contains all relevant signal representations and derived features, while the associated metadata provides sufficient contextual information to facilitate reproducibility and further analysis.

**Fig. 2:**
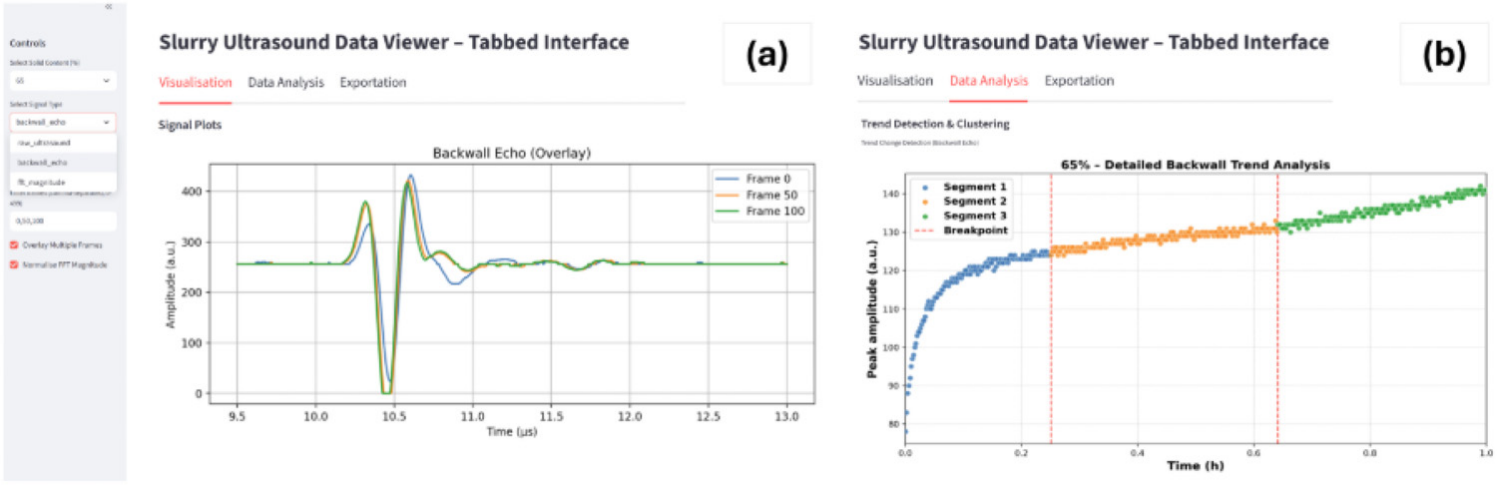
Screenshot of the Streamlit Slurry Ultrasound Data Viewer with a tabbed interface, showing (a) signal visualisation (backwall echo overlay for multiple frames) on the left and (b) data analysis on the right.

A set of lightweight Python scripts has been provided for efficient exploration and analysis of the dataset. One script allows users to load and visualise A – scans, including raw ultrasound signals, backwall echoes, and FFT magnitude spectra as seen in Fig. 1 -c. Separate scripts implement trend change detection on backwall echo time – domain signals and clustering on FFT signals. In addition, a Streamlit graphical interface integrating these functionalities, as seen in Fig. 2, enabling users to load and visualise data, select single or multiple frames, apply analyses, and export results to Excel is also provided. This workflow allows rapid assessment of waveform evolution, spectral characteristics, and the influence of solid content and recovery on signal features.

In this dataset, solid content of the slurries was the only parameter systematically varied (63 – 69 wt.%) around the mostly applied solid content of 65 wt. %, while all other experimental and acquisition conditions were identical and maintained constant to eliminate their influence on the time – dependent structural recovery behaviour of the slurry during resting.

## 4 Experimental Design, Materials and Methods

A structured experimental plan was developed to generate ultrasonic measurements from NMC – 622 cathode slurries prepared at controlled solids contents (63 – 69 wt.%). Each slurry was monitored during post – mixing recovery – and, where applicable, during subsequent ageing – under static conditions. The formulation range was selected to capture practically meaningful variations around the mostly used slurry structure (65 wt.%) and processability, while repeated measurements over time enabled a consistent assessment of recovery behaviour across all conditions.

Ultrasonic monitoring was performed where we use a fixed – container measurement configuration to ensure consistent measurement geometry during the structural recovery process. All samples have put into standard container and weighted before ultrasonic scanning. The ultrasonic probe was maintained in stable contact due to the unique function of testing Rig [9] which provides repeatable testing across varying sample with the container wall using controlled coupling conditions, and acquisition parameters were kept constant throughout each experiment. This controlled setup ensured that observed signal variations primarily reflected the time – dependent evolution of slurry structure rather than measurement drift.

Slurries were produced using a standardised mixing protocol and transferred into a fixed test – container geometry (similar volume for all samples) to maintain repeatable measurement conditions as mentioned earlier. All preparation and testing were conducted at the WMG Battery Scale – Up facility, where identical handling procedures and container dimensions were used across solids contents and replicates. This approach minimised variability unrelated to formulation or time and reduced the experimental degree of freedom.

Ultrasonic measurements were obtained using a pulse – echo method with a contact transducer (5 MHz range, Evident scientific Ltd. UK branch) coupled to a pulser – receiver unit. Acquisition settings were fixed throughout, and raw A – scan waveforms were recorded over a defined time window over 1 hr during post mixing. Data collection was automated through custom scripts to ensure consistent instrument configuration and to associate each waveform unambiguously with its slurry condition (solids content, replicate identifier, and elapsed time since mixing).

For every measurement, the raw time – domain waveform is supplied together with its frequency – domain counterpart, derived via FFT. The dataset is curated in line with *Data in Brief* standards, with harmonised variable names, structured metadata detailing acquisition parameters and sample identifiers, and accompanying description files. This ensures clarity, reproducibility, and straightforward reuse of the dataset, without duplicating the analytical findings reported in the accompanying research article.

**Fig. 3:**
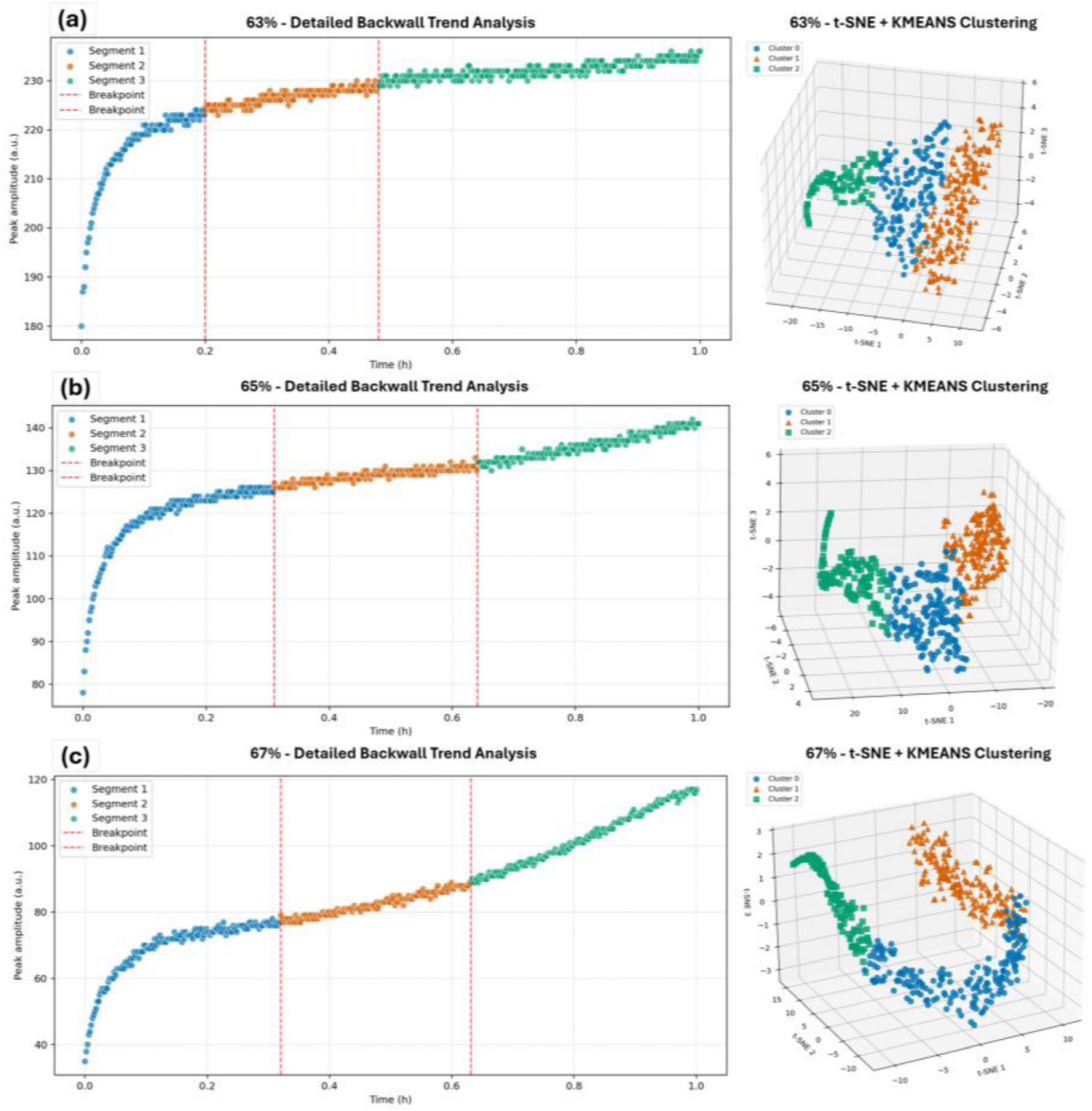
Detailed analysis of backwall signal evolution for slurry samples at 63% (a), 65% (b), and 67% (c) solids content. Left panels show trend change detection, where signals are segmented into three stages with breakpoints indicated by red dashed lines. Right panels show corresponding t – SNE visualisation of the FFT-transformed signals clustered using K – means, confirming the segmentation into three distinct regimes across all samples.

The accompanying analyses obtained via the provided python scripts and shown in Fig. 3, illustrate the utility of the dataset: backwall signal evolution for slurry samples at 63% (a), 65% (b), and 67% (c) solids content was examined using two complementary approaches. Trend change detection applied to the time – domain signals (left panels) consistently segmented each signal into three stages, with breakpoints marked by red dashed lines, capturing rapid restructuring, consolidation, and plateau phases. Independent analysis in the frequency domain (right panels) involved K – means clustering of the FFT – transformed signals, visualised using t – SNE, which confirmed the same three distinct regimes across all samples. These results demonstrate how the dataset supports both time – and frequency – domain analyses, enabling verification of signal evolution patterns and providing a foundation for further algorithm development or comparative studies.

## Limitations

A practical constraint of the dataset is the limited initial scanning time (1 hr). This is due to, once mixed, the slurry cannot be held for long without risks such as sedimentation, evaporation effects, or unintended ageing, which restricts how long continuous monitoring can be maintained under controlled conditions. The temporal coverage is therefore bounded by the realistic handling and stability limits of battery slurries.

Instrumentally, the data were obtained using a single – centre – frequency pulse – echo setup with a fixed – cup, contact – probe configuration. While repeatable, this limits the separation of attenuation, dispersion, and impedance effects, and constrains absolute velocity estimation compared with multi – frequency or through – transmission methods – especially in attenuating, heterogeneous suspensions. Here we assumed that the slurry behaves uniformly throughout the measurement volume to minimise the impact of this limitation.

Despite these limitations, the dataset includes effectively collected both raw time – domain A – scans and frequency – domain FFT outputs, enabling alternative processing approaches and robust benchmarking. Future developments could include longer – duration monitoring with improved environmental control, multi – frequency measurements, and more spatially resolved scanning to broaden its utility for modelling and process optimisation.

## Ethics Statement

The proposed data does not involve any human subjects, animal experiments, or data collected from social media platforms. The authors confirm that this work meets the ethical requirements of the journal.

## CRediT Author Statement

**Erdogan Guk:** Writing – review & editing, Writing – original draft, Conceptualisation, Visualization, Validation, Methodology, Formal analysis, Data curation. **Hamidreza Farhadi Tolie:** Writing – review & editing, Formal analysis, Data curation. **Mona Faraji Niri:** Writing – review & editing, data curation. **James Marco:** Writing – review & editing, Writing – original draft, Validation, Supervision, Resources, Project governance.

## Data Availability

Mendeley DataOpen NMC-622 Slurry Dataset: Ultrasound Measurements (Original data).
